# Immunosuppressive effect of arsenic trioxide on islet xenotransplantation prolongs xenograft survival in mice

**DOI:** 10.1038/s41419-018-0446-8

**Published:** 2018-03-14

**Authors:** Bin Zhao, Jun-jie Xia, Lu-min Wang, Chang Gao, Jia-li Li, Jia-yin Liu, Qi-jun Cheng, Chen Dai, Qi-lin Ma, Zhong-quan Qi, Ben-hua Zhao

**Affiliations:** 1 State Key Laboratory of Molecular Vaccinology and Molecular Diagnostics, Xiamen University, 361005 Xiamen, Fujian China; 2 Department of Neurology, The First Affiliated Hospital of Xiamen University, 361005 Xiamen, Fujian China; 3 Organ Transplantation Institute, Medical College, Xiamen University, 361005 Xiamen, Fujian China

## Abstract

The role of arsenic trioxide (As_2_O_3_) in inhibiting immune rejection and prolonging islet allograft survival has been identified in islet allotransplantation. This study aims to explore the role of As_2_O_3_ in islet xenotransplantation and the action mechanism. The streptozotocin (STZ) was used in C57BL/6 mice to induce the type 1 diabetes mellitus (T1DM) for xenotransplantation models establishment. Donor islets were isolated by digesting. The flow cytometry (FCM) was used to analyze lymphocyte types. The blood sugar level was detected by using intraperitoneal glucose tolerance test (IPGTT). The serum level of cytokines was determined by the enzyme-linked immunosorbent assay (ELIZA). The cell proliferation was measured by MTT assay. The mRNA levels were quantified with qRT-PCR. As_2_O_3_ prolonged the survival of the recipient mice but had no influence on body weight. As_2_O_3_ protected the function of xenograft in insulin secretion and suppressed immune rejection of recipient. As_2_O_3_ inhibited proliferation of T lymphocyte and increased the proportion of Foxp3^+^ regulatory T cells in recipient mice. As_2_O_3_ inhibited activation and promoted clonal anergy of T lymphocyte. As_2_O_3_ decreased total number of B cells and reduced partial antibody levels in recipient mice. As_2_O_3_ and leflunomide showed a synergistic effect in suppressing islet xenotransplant rejection. As_2_O_3_ prolongs islet xenograft survival by inhibiting cellular immune response, and increasing Foxp3^+^ regulatory T cells, while decreasing partial antibody levels in serum.

## Introduction

The type 1 diabetes mellitus (T1DM), also called insulin-dependent or juvenile diabetes, begins with insulin-producing beta cells attacked by autoimmune and subsequent deficiency of insulin, leading to increased glucose content in blood and urine^1^. Hyperglycemia caused by deficiency of insulin causes many complications in various tissues and organs, such as eyes, heart, blood vessels, and nerves^2^. Due to the absolute lack of endogenous insulin in T1DM, the continuous and indefinite supplement of exogenous insulin is indispensable. The main clinical treatment of diabetes mellitus includes insulin injection, oral hypoglycemic agents, combined with healthy life style, but these therapies often result in increased risk of disorder of glucose metabolism^3^. and hypoglycemia^4^. Other than exogenous substances, islet transplantation is a promising therapy for T1DM through regulating glycometabolism accordingly by blood glucose level of the organism^5^.

Recently, clinical islet transplantation for diabetes has been focused on and it has achieved success as insulin independence rates up to five years after transplant, with minimal complications^6^. Islet transplantation shows lots of advantages on simple operation, low immunosuppression, and less complication^7^, exhibiting great potential in T1DM treatment. Nevertheless, the widely use of islet transplantation is largely hampered by immune rejection and short supply of donors, especially for islet allotransplantation^8^. Islet xenotransplantation from the porcine pancreata was performed for the first time^9^, which initiated the journey of islet xenotransplantation. A consensus statement was published by the International Xenotransplantation Association (IXA) in 2009 to undertake clinical trials of porcine islet products for T1DM treatment^10^, and it was updated recently^11^, forecasting the promising clinical application of islet xenotransplantation in the near future. Islet xenotransplantation can be an attractive alternative to overcome the problem of donor shortage, but still with barriers of immune rejection, in which immunosuppressive agents are urgently needed^12^.

As a traditional drug for various disorders, arsenic trioxide (As_2_O_3_) was approved by the US Food and Drug Administration (FDA) for relapsed or refractory acute promyelocytic leukemia (APL) in 2000, after randomized clinical trials^13^. As_2_O_3_ also was reported to alleviate the allograft rejection in mice, and it inhibited allografts rejection mediated by alloreactive CD8(+) Tm cells in the mouse heart transplantation model, serving as an antirejection agent^14, 15^. In the previous study, we found that As_2_O_3_ prolonged islet allograft survival and combination of As_2_O_3_ with rapamycin showed a synergistic effect in repressing islet allotransplant rejection and inducing sustained transplant tolerance in mice^16^. These findings suggested that As_2_O_3_ is a promising and crucial immunosuppressive agent in islet allotransplant. In consideration of the limitation of islet allotransplant for donor shortage, we undertook the islet xenotransplantation from rat to mouse and explored the role and clinical values of As_2_O_3_ in islet xenotransplantation.

## Materials and methods

### Animals

The specific-pathogen-free (SPF) female Lewis rats (weight 180–200 g, 8–12 weeks old) purchased from the Beijing Vital River Laboratory Animal Technology Co. Ltd. (Beijing, China) were used as donors; SPF female C57b/6 (H-2b) mice (weight 18–20 g, 8–12 weeks old) purchased from the Slac Laboratory Animal Co. Ltd. (Shanghai, China) were used as recipients. All the animal experiments were approved by the Ethics Committee of the Xiamen University, and performed according to the guidelines of the Institutional Animal Care and Use Committee (IACUC).

### Islet isolation

Donor islets were isolated by a digestion method that described previously^17^. The pancreas was perfused with 1 mg/ml collagenase P (Roche, Basel, Switzerland) and digested at 38 °C for 20 min. After digestion, the pancreatic tissue was mixed with Histopaque-10771 and Histopaque-11191 (Sigma-Aldrich), and the purified islet cells were obtained by density gradient centrifugation.

### Flow cytometry (FCM)

Islet cells were dissociated into single cells by 0.25% trypsin-EDTA and 25 U/ml DNase I at 37 °C for 15 min, and purified lymphocytes were acquired from the spleen and inguinal lymph node after treated with the red blood cell lysis buffer and filtration. Cells were stained with monoclonal antibodies (anti-annexin V and anti-7-AAD antibodies, BD PharMingen, San Diego, CA; anti-CD4^+^, anti-CD8^+^, and anti-Foxp3^+^ antibodies, eBioscience, San Diego,CA). The results were analyzed by the BD FACScanTM system (Partec Co., Munster, Germany) and FlowJo software (Tree Star Inc., Ashland, OR).

### Diabetes induction and islet xenotransplantation

Recipient mice were treated with 180 mg/kg streptozotocin (STZ) intra-peritoneally (IP) to induce the diabetes model^18^. After three days, mice with a blood glucose level higher than 16.7 mM in two consecutive days were used for transplantation. And 400 islets were transplanted into the recipient mice under the kidney capsule and grafts with blood glucose levels less than 8.4 mM in two consecutive days were considered the successful transplantation. And it was judged as graft rejection when blood glucose level was higher than 1.1 mM in two consecutive days.

### Groups and treatments

The recipient mice were divided into four groups (*n* = 8 in each group): As_2_O_3_ group (As, 5 mg/kg As_2_O_3_ twice per day, IP), leflunomide group (Lef, 20 mg/kg Lef daily, IP), combined group (Lef + As, 5 mg/kg As_2_O_3_ twice per day and 20 mg/kg Lef daily, IP) and control group (normal saline, IP).

### Intraperitoneal glucose tolerance test (IPGTT)

The IPGTT was carried out in recipient mice at five days after transplantation. The mice were fasted for 12–14 h and then intra-peritoneally injected with blood glucose (1 mg/g). And the blood glucose levels in tail vein were detected at 0, 5, 10, 15, 30, 60, and 120 min after injection.

### Histopathology

At the 7th day after transplantation, each islet graft and the homolateral kidney were fixed in a zinc fixative (Biolegend, San Diego, CA) and embedded with paraffin, for cutting into 5-μm sections and staining with hematoxylin-eosin (H&E) or biotin-conjugated rabbit antimouse insulin monoclonal antibodies (Cell Signaling Technology Inc., Beverly, MA). The microscopy (Motic BA310; Motic Co. Ltd., Hong Kong, China) was used for image capturing. All the results were assessed in a double-blind manner.

### Quantitative real-time PCR (qRT-PCR)

Total RNA of tissues from islet graft and the homolateral kidney was extracted with trizol reagent (Invitrogen) and the cDNA was synthesized by reverse transcription with a Reverse Transcription Kit (Applied Biosystems). The expression of related genes was quantified by qRT-PCR with SYBR Select Master Mix (Applied Biosystems) on an ABI 7300-fast Real-Time PCR system. With β-actin served as control. Primers were designed and synthesized by the Sangon Biotech (Shanghai, China) and the primer sequences were showed. β-actin: forward 5′-CATCCGTAAAGACCTCTATGCCAAC-3′ and reverse 5′-ATGGAGCCACCGATCCACA-3′; IL-2: forward 5′-GGAGCAGCTGTTGATGGACCTAC-3′ and reverse 5′-AATCCAGAACATGCCGCAGAG-3′; interferon (IFN)-γ: forward 5′-CGGCACAGTCATTGAAAGCCTA-3′ and reverse 5′-GTTGCTGATGGCCTGATTGTC-3′; TGF-β: forward 5′-GACCAGCTGGACAACATACTGCTAA-3′ and reverse 5′-GATAAGGCTTGGCAACCCAAGTAA-3′; IL-4: forward 5′-CAGCTCTGCTGGCGAAAGTG-3′ and reverse 5′-TCGTCTGAAGGCAGAGTCAGGA-3′. The relative expression was calculated by the 2^-∆∆Ct^ method.

### Mixed lymphocyte reaction (MLR) and MTT assay

T lymphocytes were isolated from spleen of the recipient mice by using nylon wool columns (Wako, Osaka, Japan) at the 10th day after transplantation and served as responder cells. Spleen cells derived from Lewis rats were pretreated with mitomycin C and served as stimulator cells. MLR assays were performed as previously described^19^. After 72 h of culture, the cell proliferation was measured with a MTT cell proliferation assay kit (Cayman). Then 10 μl MTT was added into each well and maintained for 4 h at 37 °C. After the culture medium was removed, 100 μl DMSO was added and the sample was slowly agitated for 10 min for crystals dissolution at room temperature. The absorbance at 450/630 nm was measured on the microplate reader (Bio-Rad).

### ELIZA

Recipient mouse serum and MLR supernatants after 72 h of incubation were collected, and the serum level of IFN-γ, IL-2, IL-4, and TGF-β in recipient mice was determined with the enzyme-linked immunosorbent assay (ELIZA) kits (NeoBioscience Technology Co. Ltd., Beijing, China) according to the manufacturer’s instructions.

### Western blot

The expression of phosphorylated p38 (p-p38) in T lymphocytes was analyzed by western blot. For protein extraction, T lymphocytes from the recipient mice were treated with RIPA lysis buffer (Beyotime), and the concentration of protein was assessed by using a BCA protein assay kit (Beyotime). Total protein was separated with 12% SDS–PAGE and transferred onto the polyvinylidene difluoride (PVDF) membrane (Bio-Rad). The membrane was then blocked with tris-buffered saline tween-20 (TBST) containing 5% nonfat milk for 1 h at RT, and then incubated with the following primary antibodies: anti-p-p38 antibody (Abcam, 1:200), anti-β-actin antibody (Abcam, 1:3000) for 1 h at RT. The membrane was then incubated with HRP-bounded secondary antibodies for 1 h at RT and the proteins were visualized with an ECL Plus Western Blotting Substrate (Thermo Fisher). β-actin served as control to evaluate the relative expression.

### Statistical analysis

The median survival time (MST) of the animals was analyzed with the Kaplan–Meier method. Data in different groups were analyzed by the one-way analysis of variance (one-way ANOVA) and represented as means ± standard deviation (SD). Difference between groups was analyzed with Student’s *t*-test. All analyses were performed with the Graphpad Prism (GraphPad, Inc., CA) software.

## Results

### As_2_O_3_ prolonged xenografts survival and protected the function of insulin secretion

**Fig. 1:**
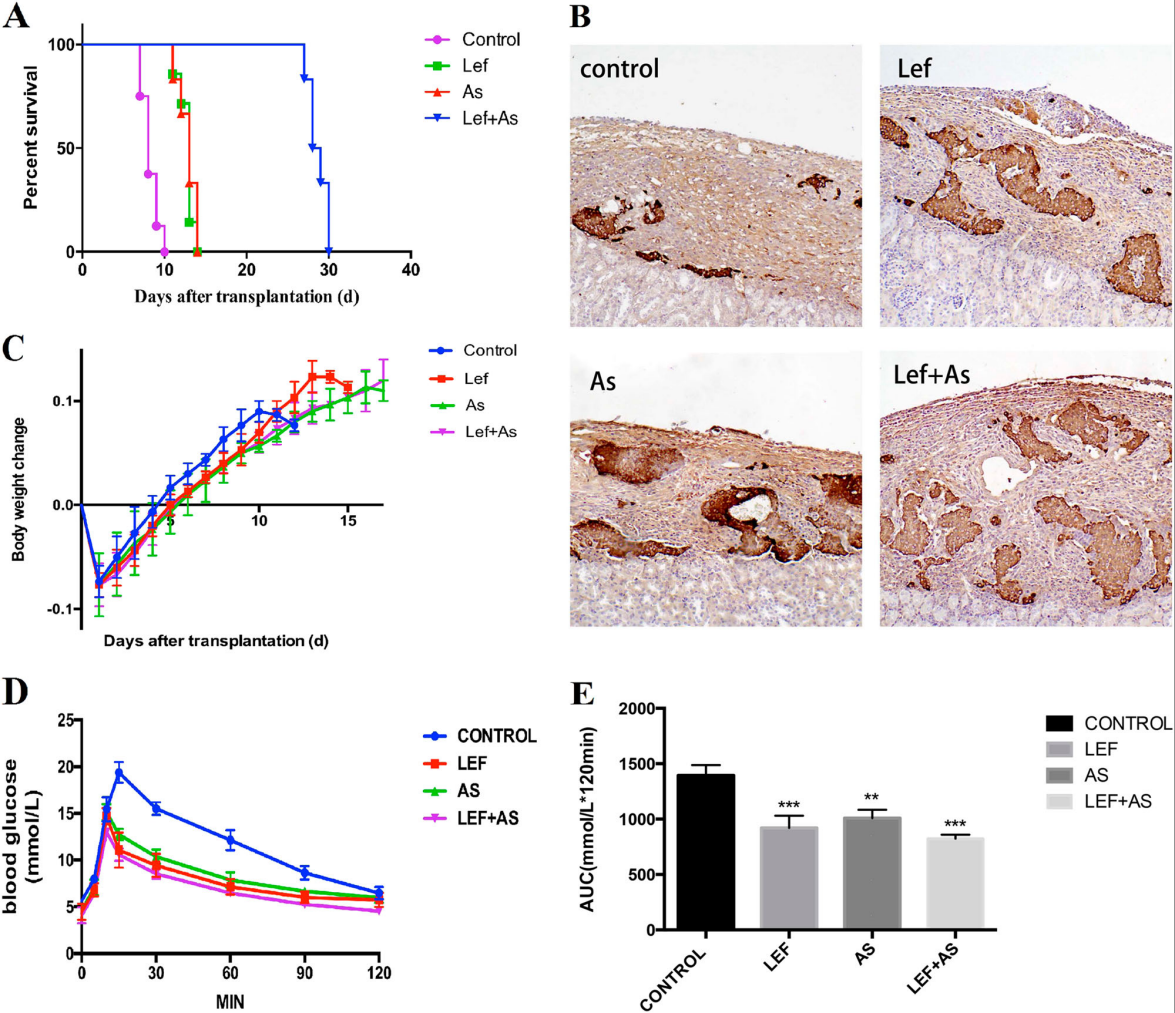
As_2_O_3_ prolonged xenografts survival and protected the function of insulin secretion. **a** Islet xenografts (Lewis to C57BL/6) survival in recipient mice with different treatment. Xenograft survival were calculated and compared by the Kaplan–Meier method. **b** Insulin immunohistochemistry assays used Paraffin-embedded sections of graft at day 7 after islet transplantation. (paraffin-embedded sections, 100× magnification). **c** Body weight changes in recipient mice with different treatments. Each data point represents the mean values of three individual mice of same treatment. **d** Blood glucose levels and **e** AUC for glucose during the IGPTT. The IGPPT was performed on 5th day after islet transplantation. Data are representative of three independent experiments. **P < 0.01 vs. control

After xenotransplantation, As_2_O_3_ (As) significantly prolonged MST of xenograft to 13 days, which was similar to the Lef group but longer than the control group (8 days); the survival time in Lef + As group (29 days) was markedly longer than that of control group, with significant difference (*P* < 0.01) (Fig. 1a). The insulin secretion condition was measured by the immunohistochemistry assay, and the insulin secretion in As and Lef + As group was better than control group, and Lef + As group showed the best insulin secretion condition (Fig. 1b). Lose weight is the main clinical symptom of T1DM, and the weight monitoring was conducted in mice before and after transplantation. It showed that the body weight of mice increased when the blood sugar maintained at the normal levels, while it declined with increased blood sugar; but no difference was found in increase trend of body weight among four groups (Fig. 1c), suggesting that body weight was little influenced by different drug treatments. The 2 h IGPTT was performed in recipient mice treated with different drugs at day 5 after transplantation, and the blood glucose levels were recorded (Fig. 1d), and the area under the curve (AUC) was statistically analyzed (Fig. 1e); and it showed that the function of islets in As, Lef, and Lef + As group was better than that of control group, and the best function of islets was observed in the Lef + As group.

### As_2_O_3_ suppressed immune rejection of recipient to xenograft

**Fig. 2:**
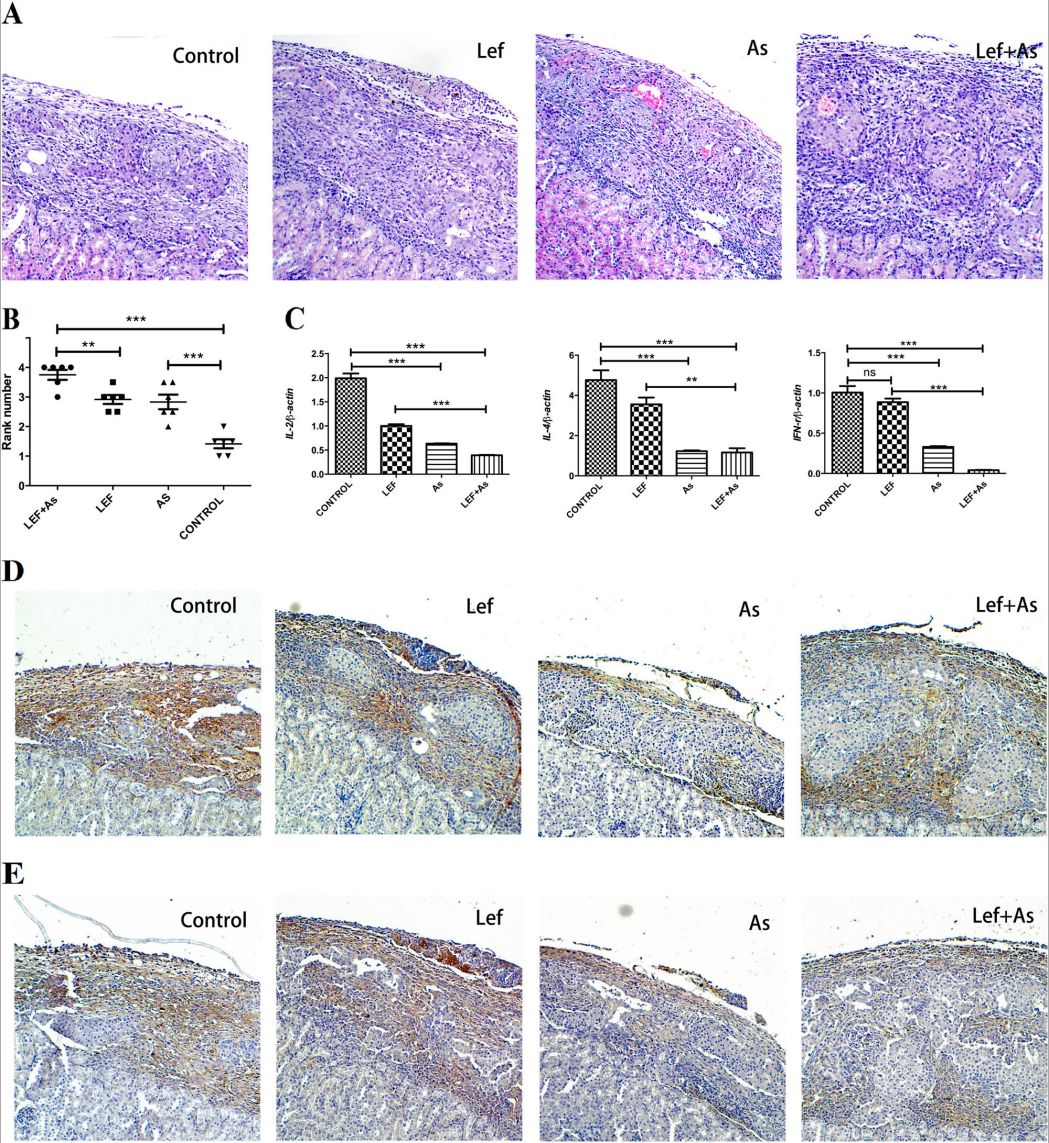
As_2_O_3_ suppressed immune rejection of recipient to xenograft. Islet xenografts were harvested on day 7 after islet transplantation. **a** H&E-stained islet xenografts. (paraffin-embedded sections, 100× magnification) and **b** the rejection scoring was used to represent the degree of infiltration of inflammatory cells. **c** The qRT-PCR was used quantified mRNA levels of IL-2, IL-4, and IFN-g within xenograft. **d** CD4^+^ T lymphocytes and **e** CD8^+^ T lymphocytes immunohistochemistry assays used Paraffin-embedded sections of graft at day 7 after islet transplantation. Data shown was the mean ± SD and is representative of three separate experiments. **P < 0.01 vs. control; ***P < 0.001 vs. control

At the 7th day after transplantation, the conditions of tissue destruction and inflammatory infiltration were evaluated by H&E staining (Fig. 2a), with a pathological score (rank number). Recipient mice in the Lef + As group showed the highest pathological score, and the scores in Lef and As group were all significantly higher than that in control group, which had the lowest pathological score (Fig. 2b). Tissues from islet graft were taken and the immune factors associated with immune rejection were quantified with qRT-PCR. Compared with control, the levels of IL-2, IL-4, and IFN-γ were obviously decreased in As and Lef + As group (Fig. 2c), suggesting that the immune rejection was suppressed by As_2_O_3_. The result of qRT-PCR was also supported by the immunohistochemistry that showed the infiltration of CD4^+^ and CD8^+^T lymphocytes. The infiltration of CD4^+^ and CD8^+^ T cells was lower in As and Lef + As group than that of control group, and it was lower in Lef + As group than that in Lef group (Figs. 2d, e).

### As_2_O_3_ inhibited proliferation of T lymphocyte

**Fig. 3:**
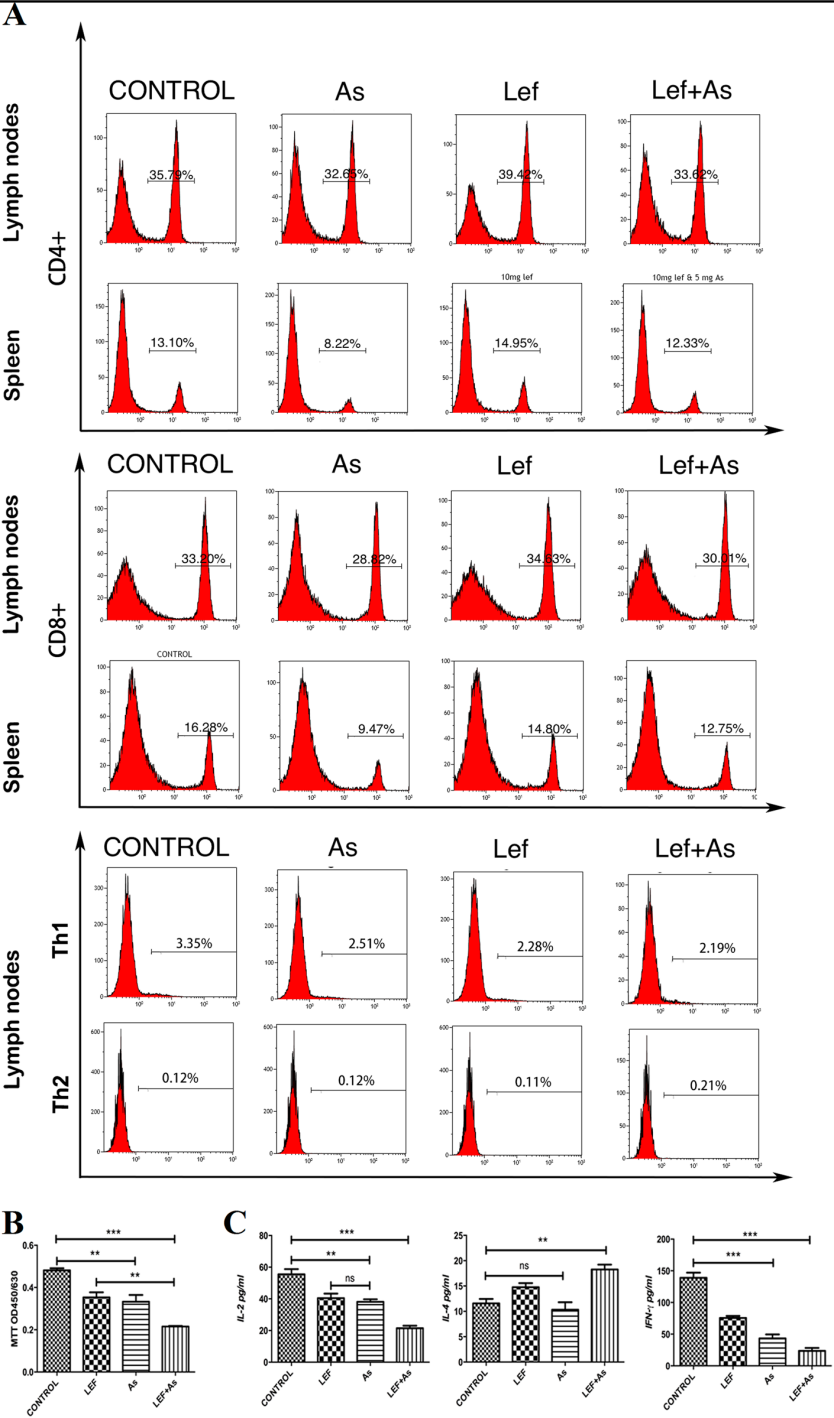
As_2_O_3_ inhibited proliferation of T lymphocyte. **a** The cumulative data of the proportion of CD4^+^ and CD8^+^T cells, and Th1 and Th2 cells in the spleen and lymph node cells of recipients were analyzed by flow cytometry on day 7 after transplantation. **b** Seven days after transplantation, MTT were used to examine the proliferative response of recipient (C57BL/6) spleen T cells to donor-type spleen cell (Lewis). **c** ELIZAs were used to test the concentrations of cytokine in the recipient serum collect on day 7 post transplantation. Each group was tested in quadruplicate wells. The shown percentages are only indicative of the representative data of three separate experiments. **P < 0.01 vs. control; ***P < 0.001 vs. control

At the 7th day after transplantation, cell typing of lymphocytes in the spleen and lymph node cells was conducted with FCM. Compared with control group, As_2_O_3_ reduced the proportion of CD4^+^ and CD8^+^ T cells, regardless of whether Lef was used, and their percentage in Lef group was slightly higher than that of control group; the proportion of Th1 cells in As and Lef + As group was lower than that of control group, and the proportion of Th2 cells in groups was too low to compare with each other (Fig. 3a). The proliferation activity of lymphocytes was determined by MTT assay and the levels of serum cytokines derived from Th1 and Th2 cells were detected by ELIZA. Compared with control group, the proliferation activity of lymphocytes was lower in As and Lef + As groups, and the Lef + As group showed the lowest proliferation activity (Fig. 3b). Compared with control group, the levels of cytokines derived from Th1 cells (IL-2 and IFN-γ) were lower in As and Lef + As groups, which was the lowest in combination group. The levels of cytokines derived from Th2 cells (IL-4) in combination group were higher than that in control, while no significant difference was noted between As and control group (Fig. 3c).

### As_2_O_3_ inhibited activation and promoted clonal anergy of T lymphocyte

**Fig. 4:**
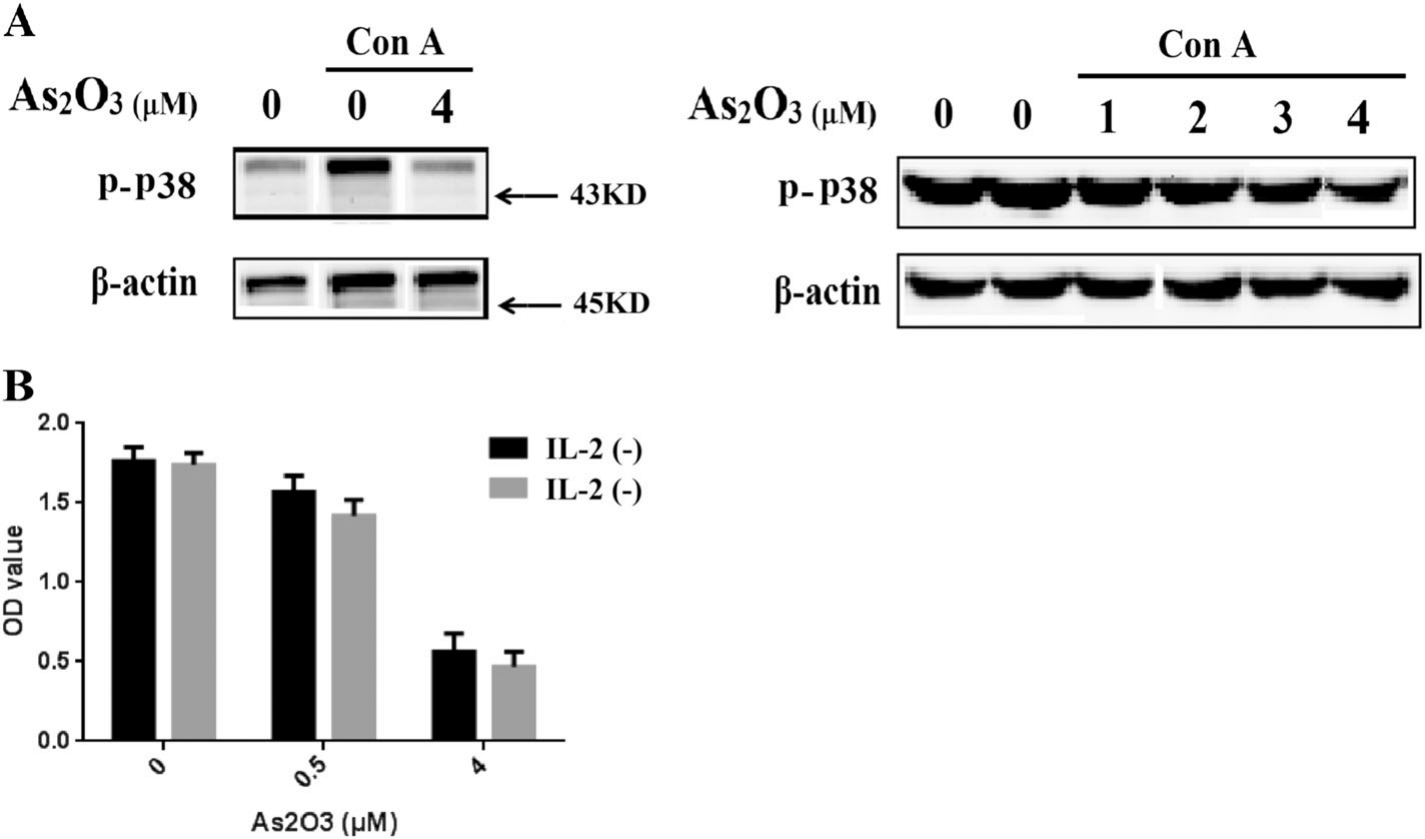
As_2_O_3_ inhibited activation and promoted clonal anergy of T lymphocyte. **a** After induced by Concanavalin A (Con A, 5 μg/ml) and treated with As_2_O_3_ at different concentrations for 24 h, the expression of phosphorylated p38 (p-p38) was determined by western blot. **b** With exogenous IL-2 added, T lymphocytes from the recipient mice were treated with Con A (5 μg/ml) and As_2_O_3_ at different concentrations for 48 h, and the proliferation activity was examined with MTT

In our previous study, we identified that As_2_O_3_ combined with rapamycin suppressed T cell activation via inhibiting p38-MAPK signaling pathways^16^. Here, to evaluation the influence of As_2_O_3_ on T lymphocyte activation, T lymphocyte was induced by Concanavalin A (Con A, 5 μg/ml) for proliferation, and simultaneously treated with As_2_O_3_ at different concentrations for 24 h. And the expression of p-p38 was determined. It revealed that As_2_O_3_ inhibited the phosphorylation of p38 in T lymphocyte, with a dosage-dependent method (Fig. 4a), suggesting the inhibitory effect of As_2_O_3_ on T lymphocyte activation. Besides, Interleukin-2 (IL-2) is a cytokine that stimulates proliferation and differentiation of T lymphocytes, and it induces the secretion of IFN-γ by T lymphocytes as well. With exogenous IL-2 added, T lymphocytes from the recipient mice were treated with Con A (5 μg/ml) and As_2_O_3_ at different concentrations for 48 h. It showed that exogenous IL-2 could not reverse the proliferation suppression induced by As_2_O_3_, implying the role of As_2_O_3_ in promoting clonal anergy of T lymphocyte (Fig. 4b).

### As_2_O_3_ increased the proportion of Foxp3^+^ regulatory T cells in recipient mice

**Fig. 5:**
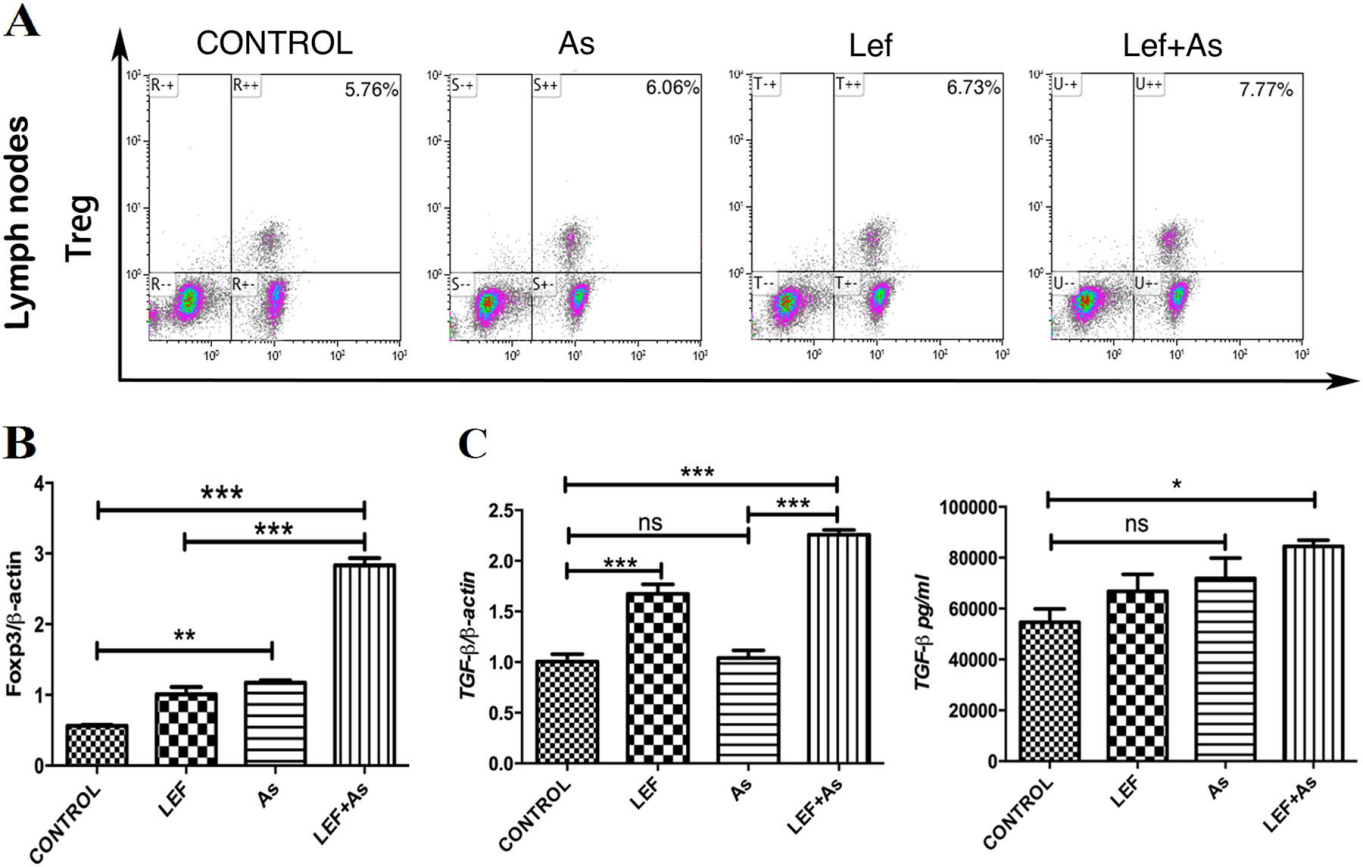
As_2_O_3_ increased the proportion of Foxp3^+^ regulatory T cells in recipient mice. **a** Proportions of splenic and lymph node CD4^+^ Foxp3^+^ Treg cells were analyzed by flow cytometry at day 7 after transplantation (*n* = 3 mice per group). **b** Graft TGF-beta and Foxp3 mRNA levels were quantified by qRT-PCR, and **c** recipient mouse sera TGF-beta concentrations were quantified by ELIZA. ELIZAs were used to test the concentrations of TGF-beta in the recipient serum collect on day 7 after transplantation. Each group was tested in quadruplicate wells. The shown percentages are only indicative of the representative data of three separate experiments. *P < 0.05 vs. control; **P < 0.01 vs. control; ***P < 0.001 vs. control

The detection of CD4^+^ Foxp3^+^ T regulatory (Treg) cells in lymph nodes indicated that the percentage of Treg cells was higher in As and Lef + As group than that in control group, and the combination group showed the highest Treg cells proportion (Fig. 5a). The Foxp3^+^ expression was higher in As and Lef + As group than that in control group, and it was highest in the combination group (Fig. 5b). Similarly, the expression of TGF-β was lowest in control and highest in the combination group (Fig. 5c).

### As_2_O_3_ decreased total number of B cells and reduced partial antibody levels in recipient mice

**Fig. 6:**
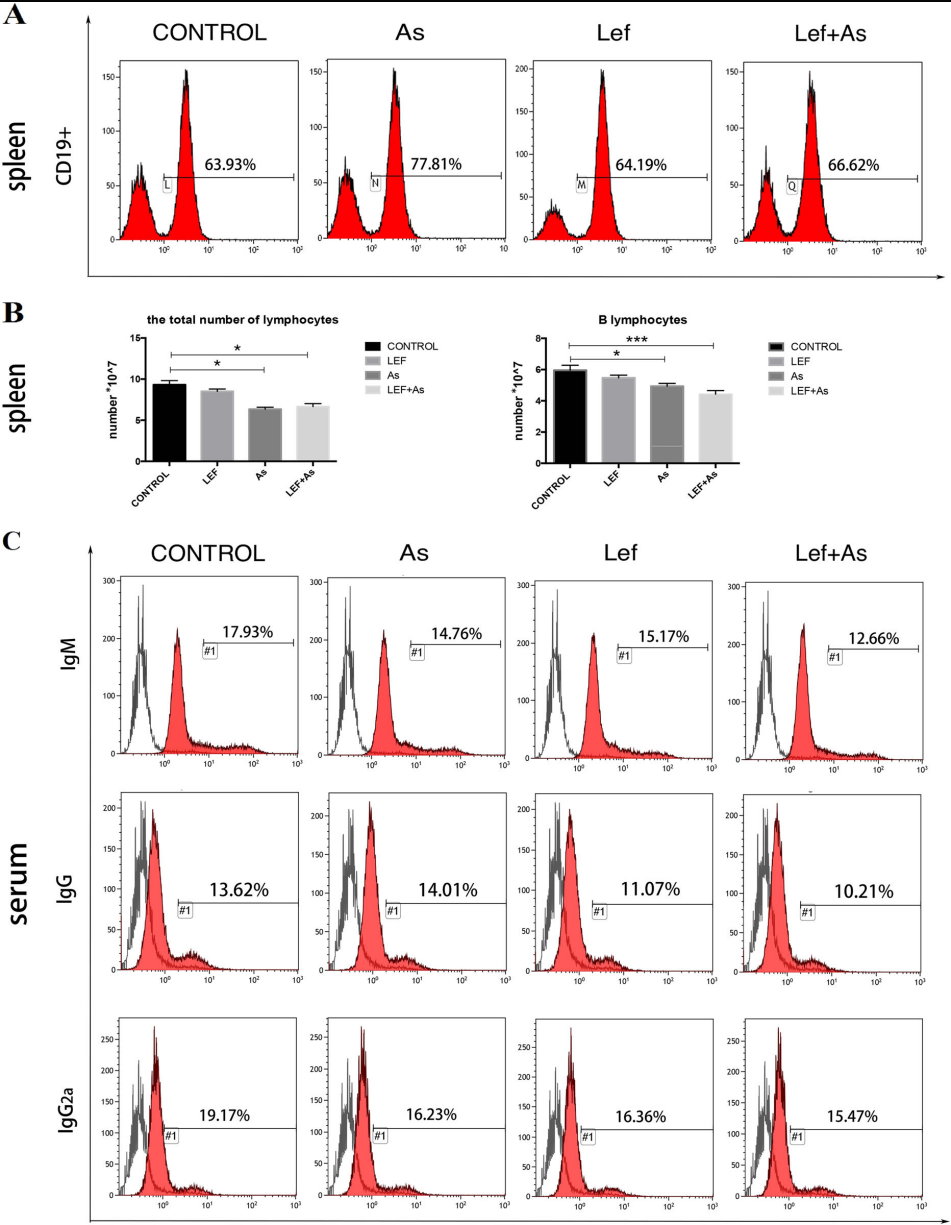
As_2_O_3_ decreased total number of B cells and reduced partial antibody levels in recipient mice. **a** Proportions of splenic and lymph node CD19^+^ B cells were analyzed by flow cytometry at day 7 after transplantation (*n* = 3 mice per group). **b** The total number of lymphocytes and B lymphocytes in spleen. **c** Proportions of IgM, IgG, and IgG2a in recipient mouse sera were analyzed by flow cytometry at day 7 after transplantation (*n* = 3 mice per group). *P < 0.05 vs. control; ***P < 0.001 vs. control

The proportion of CD19^+^ B cells in spleen of recipient mice was examined, and it showed that the percentage of CD19^+^ B cells in As and Lef + As group was significantly higher than that of control group, and it was the highest in As group (Fig. 6a). The total number of lymphocytes and B cells in spleen in As and Lef + As groups was lower than that of control, and the total number of lymphocytes was the lowest in As group and the B cells were the least in combination group (Fig. 6b). The levels of serum antibodies related to immune rejection were detected. The levels of IgM and IgG2a were lower in As and Lef + As groups, and the combination group had the lowest antibody levels, while IgG levels in As group was not lower than that in control group (Fig. 6c).

## Discussion

With the Edmonton protocol for islet transplantation proposed in the New England Journal of Medicine^20^, islet xenotransplantation has become a popular research topic once more. Islet transplantation involves in transplantation of cell clusters, with the feature of none angiogenic cerebral immune rejection from donor and inability of single donor transplantation, which enables islet xenotransplantation to be a cross hot issue between xenotransplantation and islet transplantation^21, 22^. Nevertheless, the mechanism of refection in islet xenotransplantation is extremely complicated and no successful immune tolerance induction in xenograft has been reported yet. In this study, As_2_O_3_ was proved to be an effective immunosuppressant that could be used alone in islet xenotransplantation from rat to mouse. It prolonged islet xenograft survival and protected islet transplant from inflammation and T cells infiltration to maintain the normal shape, structure, and function of insulin secretion. In addition, As_2_O_3_ had no influence on body weight of recipent mice at the dose of 5 mg/kg/d.

Rejection of islet xenografts is an immune response that closely associated with T cells^23^, which can be divided into variety of subpopulations. Among them are the CD8^+^ and CD4^+^ T cells, and they are the predominant T cells for graft-infiltrting in graft rejection^24^. It has been revealed that activated CD8^+^ T cells infiltrated cardiac allografts continuously within 72 h after transplantation, in which they promoted graft rejection via producing high levels of proinflammatory IFN-γ^25^, and the production of IFN-γ by CD8^+^ T cells has been speculated to be the rate-limiting step in process of allograft rejection^26^. And the CD4^+^ T cells has been demonstrated to be necessary and sufficient for cardiac allograft rejection, in which the intact host major histocompatibility complex (MHC) Class II is required^27^. Besides, the protective potential of CD4^+^ CD25^+^Foxp3^+^ Treg cells has been identified in xenotransplantation^28^. According to the findings of the present study, we can believe that As_2_O_3_ significantly inhibited the cellular immunity level by reducing the proportion of CD4^+^ and CD8^+^ T cells, and decreasing the proportion of Th1 cells in CD4^+^ T cells, and elevating the proportion of CD4^+^ Foxp3^+^ Treg cells. As_2_O_3_ also decreased the absolute number of B cells in spleen and lessened the level of IgM and IgG2a, but it had no effect on levels of IgG, which plays key role in humoral immunity. And the levels of humoral immunity didn’t differ significantly among different treatment groups, indicating that the influence of As_2_O_3_ on humoral immunity should be further studied.

Combination of As_2_O_3_ and Lef was more effective than individual of the both in prolonging islet xenograft survival and in various index in vivo and in vitro, suggesting the synergistic effect between As_2_O_3_ and Lef in immunosuppressive process. As a low molecular weight immunosuppressive drug, Lef inhibited the activity of dihydroorotate dehydrogenase and protein tyrosine kinase, which are the vital participants in immune reaction^29^. However, long-term of transplantation tolerance was not achieved by combination of As_2_O_3_ and Lef, which may result from the unsatisfactory inhibitory effect of Lef on B cells. Therefore, combination therapy that suppresses B cells effectively should be proposed to realize better immune tolerance between recipent mice and transplantaiton. In addition, influence of As_2_O_3_ on Th2 cells was not determinted in our study, which plays crucial role in humoral immunity and activation of B cells^30^. We speculated that it may be unsuitable for Th2 cells to be detected at day 7 after transplantation, at which the Th2 may have not reach a peak. In the future research, the variation trend and role of Th2 in islet transplantation may be explored at different time points to get more information.

In conclusion, As_2_O_3_ prolongs islet xenograft survival by inhibiting cellular immune response, increasing Foxp3^+^ regulatory T cells and decreasing partial antibody levels in serum, and it showed a synergistic effect with leflunomide in immunosuppressive effect. This study confirmed the pivotal role and great clinical values of As_2_O_3_ in islet xenotransplantation, providing significant theoretical foundation for clinical application of islet xenotransplantation.
